# Nutritional and Non-Nutritional Predictors of Low Spot Urinary Creatinine Concentration in Patients with Heart Failure

**DOI:** 10.3390/nu13113994

**Published:** 2021-11-09

**Authors:** Jolanta Malinowska-Borowska, Aleksandra Kulik, Marta Buczkowska, Weronika Ostręga, Apolonia Stefaniak, Małgorzata Piecuch, Jagoda Garbicz, Jolanta Urszula Nowak, Mateusz Tajstra, Ewa Anita Jankowska, Mariusz Gąsior, Piotr Rozentryt

**Affiliations:** 1 Department of Toxicology and Health Protection, Faculty of Health Sciences in Bytom, Medical University of Silesia in Katowice, 41-902 Bytom, Poland; akulik@sum.edu.pl (A.K.); mbuczkowska@sum.edu.pl (M.B.); weronika.ostrega@gmail.com (W.O.); apolonia.stefaniak@gmail.com (A.S.); piegosia@gmail.com (M.P.); jagoda.garbicz@gmail.com (J.G.); prozentryt@sum.edu.pl (P.R.); 2 Department of Cardiology, Faculty of Medical Sciences in Zabrze, Silesian Centre for Heart Disease, Medical University of Silesia, 41-800 Zabrze, Poland; nowjola@wp.pl (J.U.N.); mateusztajstra@wp.pl (M.T.); m.gasior@sccs.pl (M.G.); 3 Department of Heart Diseases, Faculty of Health Sciences, Wroclaw Medical University, 50-556 Wrocław, Poland; evitajankowska@gmail.com

**Keywords:** spot urinary creatinine concentration, nutritional predictors, non-nutritional predictors, heart failure

## Abstract

Low spot urinary creatinine concentration (SUCR) is a marker of muscle wasting and clinical outcome. The risk factors for low SUCR in heart failure (HF) remain poorly understood. We explored the risk factors for low SUCR related to poor outcomes. In 721 HF patients (age: 52.3 ± 11 years, female: 14%, NYHA: 2.7 ± 0.7) SUCR and Dexa body composition scans were performed. BMI prior HF-onset, weight loss, and appendicular muscle mass were obtained. Each patient was classified as malnutrition or normal by GLIM criteria and three other biochemical indices (CONUT, PNI, and GRNI). Sarcopenia index (SI) as creatinine to cystatin C ratio was also calculated. Within 1 year, 80 (11.1%) patients died. In ROC curve we identified a SUCR value of 0.628 g/L as optimally discriminating surviving from dead. In low SUCR group more advanced HF, higher weight loss and catabolic components of weight trajectory (CCWT), more frequent under-nutrition by GLIM, and lower SI were observed. In multivariate analysis the independent predictors of low SUCR were SI, CCWT, and GNRI score. In conclusion: the risk of low SUCR was associated with a worse outcome. Low SUCR was associated with greater catabolism and sarcopenia but not with biochemical indices of malnutrition.

## 1. Introduction

Heart failure (HF) has become an epidemic with the number of affected exceeding 64 million worldwide [1]. Despite modern therapy and some improvements in prognosis, still the morbidity and mortality associated with HF remains unacceptably high [2,3]. There is an increasing awareness among medical professionals that HF is a complex multiorgan syndrome with a significant contribution of noncardiac diseases in the overall risk [4]. The reciprocal interaction between various organs is the key issue. Well-known cross-talk between the heart and skeletal muscles stands behind the development of myopathy, which is one of the most important factors affecting symptoms and prognosis [5].

Phosphocreatine is a central compound of energy metabolism, serving to regenerate adenosine triphosphate from the product of its hydrolysis–adenosine diphosphate [6]. Skeletal muscle is the main organ where phosphocreatine is broken down to its final waste product—creatinine. Apart from the use of serum creatinine as a marker of kidney function, recent studies point out the utility of its 24-h urinary excretion as a surrogate marker of skeletal muscle mass and performance [7]. Furthermore, it was shown that a low urinary excretion rate of creatinine is strongly associated with a poor prognosis in various diseases [8,9,10,11,12], including heart failure [13].

Although useful, measuring the urinary excretion rate by timely urine collection can be cumbersome in clinical practice, especially in elderly women. Replacement of timely urine collection with spot urinary creatinine (SUCR) concentration would overcome at least part of these limitations. Two recent studies have shown the association of low SUCR with muscle wasting and with poor prognosis in HF patients [14,15]. However, the risk factors for SUCR that clearly increase mortality risk are not identified yet. Of particular interest is the association between low SUCR and nutritional parameters because muscle wasting is linked to urinary excretion of creatinine and both strongly related to under-nutrition [16]. Under-nutrition denotes insufficient intake of energy and nutrients to meet an individual’s needs to maintain good health. In literature, under-nutrition is used synonymously with malnutrition [17]. There is no common consensus regarding the most useful tool to assess the nutritional status. For that reason, Controlling Nutritional status (CONUT) score, Prognostic Nutritional Index (PNI), Geriatric Nutritional Risk Index (GRNI), Sarcopenia index and Glim criteria were used in this study. To our knowledge, no studies so far addressed risk factors of SUCR range that is clearly associated with poor outcome.

Therefore, the aim of the study was to explore the independent predictors of low SUCR with particular emphasis on biochemical, easy-to-obtain markers of malnutrition as contributing factors.

## 2. Materials and Methods

### 2.1. Study Group

Data collected in the Prospective Registry of Heart Failure implemented in our Department in 2003 were used in this study. Patients with HF and reduced left ventricle ejection fraction (LVEF) ≤ 40%, diagnosed according to criteria published by the European Society of Cardiology, aged > 18 years and with HF duration of more than 6 months, recruited in outpatient settings from January 2004 to March 2013, on the best tolerated medical therapy, for whom HF could be confirmed with 1 month precision and with available records concerning body weight before the first diagnosis of HF and minimal weight during HF were selected.

The onset of HF was defined as a month in which medical records prepared by a cardiologist on an outpatient demonstrated the coexistence of LVEF ≤ 40% with typical signs and/or symptoms of HF. Maximal unchanged therapy had to be longer than 1 month before the index date. The maximal body weight was defined based on the outpatient medical records as the highest weight within a year, but not later than 2 months before HF diagnosis. Conversely, the lowest body weight was defined as the minimum congestion-free body weight, when the attending cardiologist in a clinical examination did not change diuretics or did not note signs and/or symptoms of fluid retention.

Patients treated with glucocorticosteroids, bisphosphonates, vitamin D supplements, or calcium or phosphorus salts; those having active infection, liver disease with enzymes levels four times higher than normal, active bleeding, known neoplasm, or granulomatous disease and those who had undergone bariatric surgery or surgery reducing intestinal absorptive capacity were excluded. Out of 1102 registry participants, 721 fulfilled the study criteria of having performed SUCR analysis and DEXA scanning. Medical records of this study group were reviewed, and comorbidities such as hypertension, diabetes mellitus, and hypercholesterolaemia were recognized based on clinical history, current medication, or actual measurements of the respective variables. History of smoking was defined as current or previous use of tobacco products.

### 2.2. Methods

One spot urine sample was collected per person on the index day. Blood samples were drawn in a standardized manner in the morning, between 8 and 10 AM, from patients who had been fasting for at least 8 h and resting in a supine position in a quiet, environmentally controlled room for 30 min. Blood was immediately centrifuged in 4 °C and stored at −75 °C for further analyses. All procedures were undertaken in accordance with Helsinki Declaration. The protocol was reviewed and accepted by the Ethical Committee of Medical University of Silesia in Katowice (NN-6501-12/I/04). All patients expressed their informed, written consent.

Body mass and height were measured on the day of blood sampling (index date) using a certified scale (B150L, Redwag, Zawiercie, Poland). Body mass index (BMI) was calculated by dividing weight in kilograms by height in meters squared. Only index weight was directly measured. PreHF and minimal HF body weights were obtained from medical records as described above. PreHF BMI, minHF BMI, and index BMI corresponding to maximal, minimal, and index weights were defined in this study. According to our previously described concept, we have calculated four indices reflecting the oedema-free weight trajectory from HF onset until index date [18]:

1. Weight loss [%] = 100 × (preHF BMI-index BMI)/preHF BMI;
2. Catabolic component = 100 × (minHF BMI–preHF BMI)/preHF BMI, (negative value or zero if minHF BMI = preHF BMI);
3. Anabolic component = 100 × (index BMI–minHF BMI)/minHF BMI, (positive value or zero if index BMI = minHF BMI); and
4. Catabolic/anabolic balance = Catabolic component − anabolic component.

Sonos-5000 Hewlett-Packard Ultrasound Scanner (Hewlett-Packard, Andover, MA, USA) was used to measure LVEF from the apical four-chamber view and calculate it with the following formula:

LVEF = [(end-diastolic volume-end-systolic volume)/end-diastolic volume] × 100

Body composition analysis was performed with the use of dual X-ray absorptiometry (DEXA) with a pencil beam Lunar DRX-L device (General Electric, Brussels, Belgium). Compartments of body mass were measured and used in further analyses. Commercially available reagents (Roche Diagnostics, Basel, Switzerland) allowed to measure serum creatinine, sodium, N-terminal pro-brain natriuretic peptide (NTproBNP), serum albumin, haematology, hemoglobin and number of lymphocytes, hCRP, and cystatin C. eGFR was calculated from MDRD formula: eGFRMDRD = 186 × plasma creatinine [mg/dL] − 1.154 × age [years] − 0.203 × 0.742 (if female).

Five nutritional indices were used in the study:

1. Controlling Nutritional Status (CONUT). The categories of normal nutrition and different levels of under-nutrition were calculated using serum albumin, cholesterol, and number of lymphocytes [19]. For the purpose of our study, we combined different stages of under-nutrition into one category.
2. Prognostic Nutritional Index (PNI) [20] was calculated taking advantage of the formula: 10 × serum albumin (g/dL) + 0.005 × lymphocyte count (mm^3^). A score > 38 was considered normal, while patients with scores < 35 were categorized as under-nutrition.
3. Geriatric Nutritional Risk Index (GRNI) was calculated based on the formula: 1.489 × serum albumin (g/L) + 41.7 × (body weight in kilograms/ideal body weight) [21]. The ideal body weight was calculated using the formula: 22 × square of height in meters [22]. A score > 98 was considered normal; scores below 98 were considered as the under-nutrition.
4. Sarcopenia index defined as 10 × (creatinine [mg/dL]/cystatin C mg/dL) [23].
5. GLIM (Global Leadership Initiative on Malnutrition) criteria for recognition of undernutrition use a combination of etiologic criteria, in our case it was HF, with at least one of three of the phenotypic criteria: weight loss exceeding 10%, BMI < 20 kg/m^2^ before the onset of etiologic factor in patients younger than 70 years or <22 kg/m^2^ in older people, and muscle mass loss assessed with validated technology and defined cut-offs. For our current study, we used the height-indexed appendicular skeletal muscle mass (ASMI) detected by DEXA scanning and applied the cut-offs recommended in the revision of the consensus of the European Working Group on Sarcopenia in Older People [24]. The cut-offs of ASMI were <7.0 kg/m^2^ in men and <5.5 kg/m^2^ in women.

### 2.3. Statistics

Continuous variables are presented as mean values and standard deviations, categorical as percentages. Non-normally distributed data (Shapiro–Wilk testing) are presented as median and interquartile ranges (IQR). Separate logistic regression analyses were conducted and adjusted for relevant covariates of SUCR. Later a manual stepwise backward elimination was performed based on *p*-values ≤ 0.05 for variable selection. In order to select variables on adjusted odds ratios, the covariates were locked into the multivariable logistic regression model. The predictive performance of the final model was evaluated by receiver-operating characteristic (ROC) curves. The study group was split into SUCR values identified by receiver operating analysis as optimally discriminating patients who survived from those who died within 1 year of follow-up. The cut-off point was obtained using Yuden index method. This value allowed splitting the study cohort into groups below and above the cut-off point. The Kaplan–Meier survival curves was constructed to ensure that the cut-off identifies patients with different risks.

Clinical, laboratory, body composition, nutritional, and other characteristics were compared between groups using Student *t*-test, Mann–Whitney U-test or chi-square tests where appropriate.

Later, we took advantage of univariate logistic regression to identify the risk of lower compared to higher cut-off of SUCR for all characteristics studied. Furthermore, a multiply logistic regression analysis was performed to estimate the risk of lower SUCR by fitting variables with *p* < 0.05 in univariate analysis. Two models were computed. The first included GLIM, but not the lean mass and parameters characterizing weight trajectory as they are aggregated in GLIM. In the second analysis, we replaced GLIM by these characteristics. For all analyses, the significance level was set at 0.05 (two-tailed) and all calculations were performed using the software package of Statistica v.13 (Statsoft, Poland).

## 3. Results

**Figure 1:**
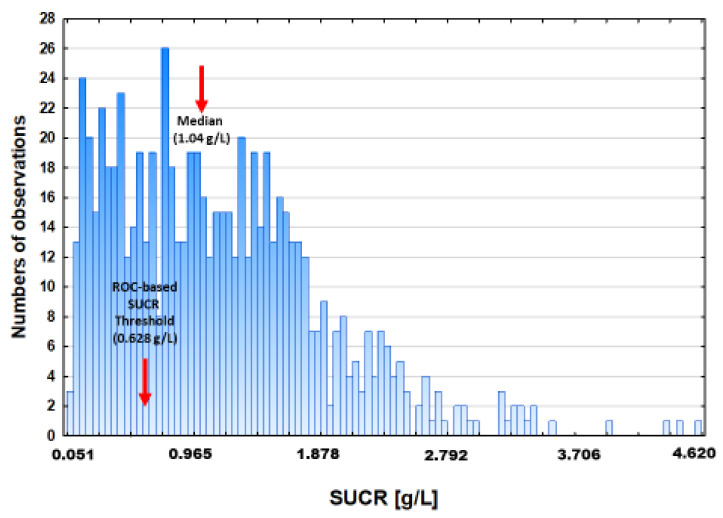
The distribution of SUCR in the study cohort and cut-off point identified by ROC analysis (N = 721).

The study cohort counted of 721 HF patients, mean age: 52 ± 11 years, 86% male, NYHA class: 2.7 ± 0.7, 57% ischemic aetiology. Detailed characteristics regarding clinical, laboratory, body composition, nutritional, and biochemical characteristics are provided in Table 1. The distribution of patients across SUCR values was highly skewed with a predominance of patients far below the median (Figure 1).

The study group was split according to SUCR cut-off value identified using ROC analysis of 1 year all-cause mortality data. During the follow-up, 80 (11.1%) patients died. The calculated area under receiver operating characteristics for SUCR was 0.580; (95%CI: 0.523–0.632), *p* = 0.006, and the optimal cut-off separating dead from alive was 0.628 g/L. The division of the study cohort according to this point produced a group of 221 (30.7%) patients falling below and 510 (69.3%) above the SUCR threshold (Table 2).

The comparison of these groups has shown that patients with low SUCR had more advanced HF stage as evidenced by worse NYHA class, lower systolic blood pressure, higher levels of NTproBNP and lower serum sodium. Consequently, the history of their weight changes during HF also reflected a more severe illness. They lost more body weight expressed as a percentage of the initial BMI. They had a larger catabolic component of weight trajectory and a strong trend toward a more catabolic profile of catabolic/anabolic balance. As a result of these alterations, their index BMI was also lower (Table 2).

Body composition markers also varied between groups. In patients with low as compared to high SUCR, the global fat-free mass showed a strong trend toward lower values, while in the case of ASMI, either the means or the percentage of patients with reduced ASMI were significantly lower in this group (Table 2).

Apart from differences in NTproBNP and serum sodium, the biochemistry profile of study groups was similar. In addition, all nutritional indices, except sarcopenia index and GLIM, were similar. The sarcopenia index was lower and the under-nutrition by GLIM index was more prevalent in a group with lower SUCR. Neither the prevalence of comorbidities nor the therapy instituted for the patients was different.

**Figure 2:**
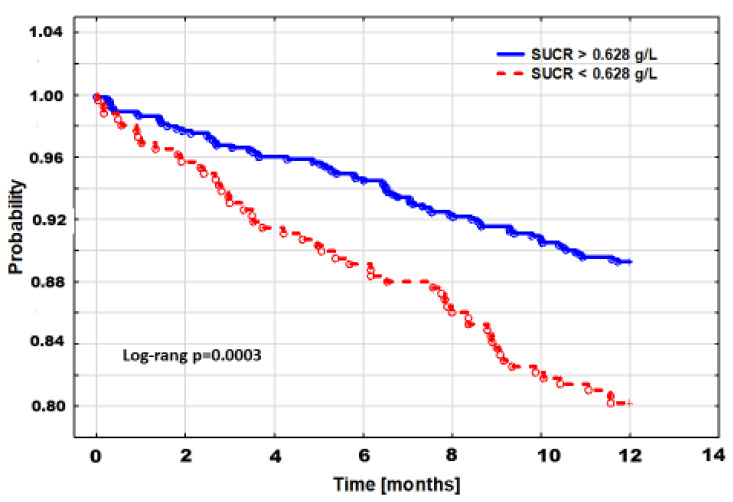
Kaplan–Meier cumulative survival curves for low and high SUCR.

According to their different clinical pictures, the 1-year probability of death was clearly higher in patients with low SUCR compared to those with SUCR above the threshold. This difference was graphically confirmed by Kaplan–Meier cumulative probability of survival (Figure 2).

In univariate logistic regression analysis, we were able to identify several predictors of low SUCR (Table 3). It should be noted that only GLIM classification of under-nutrition was significantly associated with lower SUCR, while GNRI score as a continuous variable expressed only a trend (*p* = 0.12) for the association (Table 3). The remaining nutritional scores were not predictive of lower SUCR in univariate analysis.

We performed two separate multivariate analyses. In the first, we allowed GLIM categorical classification of under-nutrition, but excluded weight loss, catabolic, and anabolic components of weight trajectory, as well as catabolic/anabolic balance together with fat-free mass. This exclusion was based on the definition of GLIM under-nutrition, which is based on the degree of weight loss and fat-free mass. In the second analysis, we replaced GLIM categories by the variables excluded in the first analysis (Table 3).

The first multivariate analysis showed that the only independent predictors of low SUCR were the GRNI score and the sarcopenia index. For each increase in the GNRI score (meaning better nutrition), the likelihood of low SUCR decreased by 17%. On the contrary, for each unit of creatinine to cystatin C ratio, the probability of low SUCR increased by 19% (Table 3).

In the second analysis, we identified three independent predictors of low SUCR. These were: catabolic component of weight trajectory, GNRI score, and, again, creatinine to cystatin C ratio. With each 5% increase in the catabolic component, the probability of low SUCR increased by 43%. Associations of GNRI and creatinine to cystatin C ratio with lower SUCR were very similar to previous analysis with odds for lower SUCR of 0.83; (0.72–0.77), *p* = 0.02 and 1.18; (1.09–1.28), *p* < 0.0001, respectively.

## 4. Discussion

The key finding of our work is that under-nutrition as represented by GLIM classification is not the independent predictor of reduced SUCR. Similarly, biochemical indices of malnutrition taken as categorical variables are also not related to low SUCR. In contrast to previous indices, sarcopenia index significantly predicts low SUCR and its predictive power is retained independently of whether we included GLIM classification, or replaced it by more specific parameters of catabolism and body composition. The novelty of our study is also the demonstration that certain characteristic of weight change trajectory, such as its catabolic component, significantly and strongly predicts low SUCR values.

Weight loss exceeding 5% within 6–12 months, commonly called cardiac cachexia and/or muscle wasting, is a common complication of HF that occurs in up to 15% and 20% of patients, respectively [25,26]. However, the existing criteria for the diagnosis of muscle wasting or cardiac cachexia are not strictly precise and they are still debated [21,24,27]. Therefore, these distinct clinical entities frequently overlap and are recognized inter-changeably by clinicians [28]. In the current understanding of cachexia and skeletal muscle wasting in HF, under-nutrition plays a central role [29].

It is commonly accepted that facing the lack of proven therapy, the optimal strategy should be early identification of patients at risk, or diagnosis of a given abnormality at the earliest possible stage, allowing for complex intervention, including nutritional therapy [30,31].

In addition to monitoring weight changes, timely screening for risk of sarcopenia and measurement of muscle strength appear to be mandatory. However, in ambulatory settings, availability of proper dynamometers is doubtful in most units. Thus, the search for an equivalent method to monitor muscle quality and function is a hot issue.

Recently, the measurement of SUCR has attracted increasing attention because it is easily available and inexpensive. Two studies published to date were aimed at identifying determinants of SUCR and analysing the relationship between different ranges of SUCR and prognosis in HF. The populations included in these two studies differed significantly from our cohort. In both the GISSI–HF and BIOSTAT–CHF projects, patients were approximately 10 years older, between 20 and 50% of patients were not compensated, as judged by the presence of oedema or pulmonary rales, only small proportion of patients lost any weight during HF in GISSI–HF, the weight loss exceeding 5% occurred in about 20% of the patients in BIOSTAT–CHF [32]. Data on nutritional status were not provided in both studies [14,33]. There were also marked differences in terms of clinical characteristics and the rate of events between the patients included in the BIOSTAT–CHF study [33]. In the studies cited above, the follow-up was three years in contrast to one year in our study.

Despite the differences noted above, we and previous studies found that patients with lower SUCR values had a more advanced profile of HF. Consequently, the risk of death was higher in patients with lower SUCR.

In previous studies researchers focused their interest on determinants of SUCR values and used multivariate linear regression analysis as a research tool. In our study we attempted to identify independent risk factors of SUCR, especially nutritional, clearly linked to poor outcome.

Previous research suggested that in multivariate models, SUCR values were positively correlated with kidney function and hemoglobin in GISSI–HF [14], diastolic blood pressure, and serum sodium in BIOSTAT–CHF [15]. The negative correlation of SUCR was identified with urinary albumin, diabetes, age, diuretic use, NYHA class, sex, and fibrinogen levels in GISSI–HF [14] and with serum urea nitrogen, peripheral oedema above the ankles, presence of orthopnea, plasma NTproBNP, and female sex in BIOSTAT–CHF [15]. None of these determinants were independent risk factors for low SUCR in our analysis. However, due to the different analytic approaches between previous studies and our work, a direct interpretation of these discrepancies cannot be undertaken.

Despite these difficulties, an important aspect of our work is in close agreement with the results from BIOSTAT–CHF. In both studies there was a clear relationship between low SUCR and presence of weight loss, and degree of body wasting. In contrast to our work, where we were able to demonstrate higher prevalence of 10% weight loss in the low SUCR group, in BIOSTAT–CHF such a relationship was also present when more stringent Evans criteria [34] of cardiac cachexia were used. Beyond these observations from BIOSTAT–CHF, no data were available on nutrition-dependent risk factors of low SUCR.

The use of the creatinine to cystatin C ratio as a sign of low muscle mass was first proposed by Tetsuka [35], later called the sarcopenia index and validated among critically ill patients in intensive care units [36,37]. It was also clearly established that sarcopenia index is well correlated not only with muscle mass, but also with strength and markers of malnutrition and can serve as an objective tool for screening of malnutrition [23,38]. Taking into account the current diagnostic criteria for sarcopenia, these findings further support the utility of the creatinine/cystatin C ratio as a marker of sarcopenia.

In fact, several studies have shown the close association of the creatinine/cystatin C ratio with the diagnosis of sarcopenia according to contemporary guidelines [39,40,41,42]. Since some other studies failed to confirm this finding [43,44] more work is clearly needed in different populations to understand the potential pathophysiological background.

In our study, the creatinine to cystatin C ratio was significantly lower in patients with SUCR below the cut-off point. Importantly, reduced values of the sarcopenia index—suggesting the possible presence of sarcopenia—were consistently associated with a higher risk of reduced SUCR in all multivariate models.

In conclusion: Low SUCR levels are associated with a worse out-come at one year of follow-up. The levels of SUCR met in patients with higher risk are predicted by parameters linked to higher catabolism and muscle wasting. Although GLIM under-nutrition criteria are a combination of defined weight loss, low initial BMI, or low muscle wasting, the better prediction of low SUCR was provided by more specific parameters of weight trajectory and biochemical markers of muscle wasting. Among indices of under-nutrition validated among HF patients, only GRNI expressed as a score and sarcopenia index independently predicted low SUCR values.

Our study has some limitations. The main limitation of the study is a retrospective, cross-sectional design that precludes any conclusion on causality. The duration of HF from the onset to the index date was variable and data on the weight trajectory and degree of weight loss were collected at different time points in each patient. Furthermore, the information on weight before the onset of HF and on the minimum weight in HF came from medical records and/or was declared by patients and/or families. Although we paid particular attention to exclude patients with water overload, this was done only clinically without confirmation from laboratory methods, i.e., radiology, ultrasound- or intracardiac pressure measurement. The GLIM classification was based on weight loss during variable time intervals for each patient which could lead to misclassification.

## Figures and Tables

**Table 1:** Clinical and laboratory characteristics of all patients included in the study (N = 721).

Feature	Means ± Standard Deviation/Medians with 25 and 75 Percentiles or per Cent Where Appropriate
**Baseline Demographics and Functional Tests**	
Age (years)	52 ± 11
Males (%)	86
HF aetiology–ICM (%)	57
NYHA class	2.7 ± 0.7
NYHA class I/II/III/IV (%)	5/35/51/9
Duration of HF (months)	35.1; (13 ÷ 71)
Systolic BP (mmHg)	108 ± 16
Heart rate (beats per minute)	82 ± 15
MVO_2_ (mL/kg min)	15.0; (12.3 ÷ 18.4)
LVEF (%)	24 ± 7
**Anthropometrics and body composition**	
PreHF BMI (kg/m^2^)	28.3 ± 4.7
% preHF BMI < 20 kg/m^2^ if <70 years or <22 kg/m^2^ if ≥70 years (%)	2.1
IndexBMI (kg/m^2^)	26.2 ± 4.5
Weight loss from preHF BMI till index BMI (%)	7.9; (1.1 ÷ 14.3)
% with weight loss > 10%	37.1
Catabolic component of weight trajectory (%)	−11.5; (−18.3 ÷ −5.3)
Anabolic component of weight trajectory (%)	3.6; (0.0 ÷ 9.3)
Catabolic/anabolic balance (%)	−16.3; (−24.1 ÷ −10.0)
Fat mass (kg/m^2^)	7.2; (5.6 ÷ 9.0)
Fat mass (%)	27.5 ± 7.8
Fat-free mass (kg/m^2^)	17.7; (16.0 ÷ 19.4)
ASMI (kg/m^2^)	7.4 ± 1.2
ASMI < 7 kg/m^2^ if male or <5.5 kg/m^2^ if female (%)	33.7
**Nutritional indices**	
CONUT score	1.6 ± 1.4
CONUT under-nutrition present/absent (%)	49/51
PNI score	50.5 ± 5.4
PNI under-nutrition present/absent (%)	15/85
GNRI score	111.5 ± 10.7
GNRI under-nutrition present/absent (%)	10/90
Creatinine/cystatin C ratio (number)	10.37; (8.84 ÷ 12.30)
GLIM under-nutrition present/absent	34/66
**Laboratory tests**	
Hemoglobin (mmol/L)	8.7 ± 1.1
NTproBNP (pg/mL)	1474; (679 ÷ 3283)
Creatinine (µmol/L)	86; (73 ÷ 107)
eGFR_MDRD_ (mL/min × 1.73 m^2^)	85.8; (66.2 ÷ 104.1)
Sodium (mmol/L)	136; (134 ÷ 138)
hCRP (mg/dL)	2.8; (1.2 ÷ 6.7)
Spot urinary creatinine (g/L)	1.04; (0.55 ÷ 1.59)
**Comorbidities**	
Hypertension (%)	54.5
Diabetes mellitus type 2 (%)	29.4
Hypercholesterolemia (%)	60.5
Hypertriglicerydemia (%)	42.6
History of smoking (%)	73.6
**Therapy**	
ACEI/ARB (% treated)	93.9
ACEI/ARB (% of recommended dose)	50; (25 ÷ 100)
BB (% treated)	97.5
BB (% target of recommended dose)	50; (33 ÷ 67)
MRA (% treated)	95
MRA (% of recommended dose)	50; (50 ÷ 50)
Loop diuretics (% treated)	90.6
Loop diuretics (mg of furosemide eq.)	80; (40 ÷ 120)
Mortality at 1 year (%)	11.1

Legend (Table 1). ICM—ischemic aetiology, NYHA—New York Heart Association, MVO_2_—maximal oxygen consumption during treadmill exercise limited by symptoms of breathlessness, LVEF—left ventricle ejection fraction, PreHF BMI—BMI before HF onset, ASMI—Appendicular skeletal muscle index, CONUT—Controlling Nutritional Status, PNI—Prognostic Nutritional Index, GNRI—Geriatric Nutritional Risk Index, GLIM—Global Leadership Initiative on Malnutrition, NTproBNP—N-terminal propeptide of Brain-type natriuretic peptide, HsCRP—high sensitivity C-reactive protein, ACEI/ARB—Angiotensin converting enzyme inhibitor or Angiotensin receptor blocker, BB—Beta blocker, and MRA—Mineralocorticoid receptor antagonist.

**Table 2:** Clinical characteristics and comparison of subgroups defined based on SUCR thresholds optimally discriminating death patients from alive at 1 year of follow-up (N = 721).

Feature	Means ± Standard Deviation/Medians with 25 and 75 Percentiles or per Cent Where Appropriate		
	Groups of SUCR Defined Based on ROC Analysis		
	SUCR < 0.628 g/L (1 Year Mortality)		
	<0.628 g/L N = 211	≥0.628 g/L N = 510	*p*-Value
**Baseline Demographics and Functional Tests**			
Age (years)	51.4 ± 11.8	52.6 ± 10.0	0.16
Males (%)	82	87	0.08
HF aetiology–ICM (%)	57	57	0.96
NYHA class	2.7 ± 0.8	2.6 ± 0.7	0.15
NYHA class I/II/III/IV (%)	7/27/55/12	4/38/58/8	0.007
Duration of HF (months)	27; (11 ÷ 62)	38; (14 ÷ 73)	0.48
Systolic BP (mmHg)	105 ± 16	109 ± 16	0.02
Heart rate (beat per minute)	82 ± 14	82 ± 15	0.8
MVO_2_ (mL/kg min)	15.3; (12.6 ÷ 19.2)	14.9; (12.2 ÷ 18.0)	0.11
LVEF (%)	25 ± 8	24 ± 7	0.39
**Anthropometrics and body composition**			
PreHF BMI (kg/m^2^)	28.4 ± 4.9	28.3 ± 4.7	0.91
% preHF BMI < 20 kg/m^2^ if <70 years or <22 kg/m^2^ if ≥70 years (%)	3	2	0.13
IndexBMI (kg/m^2^)	25.5 ± 4.2	26.5 ± 4.5	0.01
Weight loss from preHF BMI till index BMI (%)	10.0; (4.1 ÷ 16.1)	6.6; (0.0 ÷ 13.2)	<0.001
% with weight loss > 10%	50	37	0.002
Catabolic component of weight trajectory (%)	−14.1; (−19.4 ÷ −8.8)	−10.4; (−17.6 ÷ −4.8)	<0.001
Anabolic component of weight trajectory (%)	3.2; (0.0 ÷ 8.9)	3.8; (0.0 ÷ 9.4)	0.32
Catabolic/anabolic balance (%)	−17.9; (−26.4 ÷ −11.9)	−15.0; (−23.1 ÷ −9.2)	0.06
Fat mass (kg/m^2^)	7.1; (5.1 ÷ 8.7)	7.3; (5.8 ÷ 9.1)	0.078
Fat mass (%)	27.0 ± 8.1	27.7 ± 7.7	0.27
Fat-free mass (kg/m^2^)	17.4 ± 2.5	17.8 ± 2.7	0.06
ASMI (kg/m^2^)	7.3 ± 1.2	7.5 ± 1.2	0.04
ASMI < 7 kg/m^2^ if male or <5.5 kg/m^2^ if female (%)	38	32	0.09
**Nutritional indices**			
CONUT score	1.67 ± 1.4	1.65 ± 1.4	0.84
CONUT under-nutrition present/absent (%)	51	49	0.68
PNI score	50.8 ± 5.7	50.4 ± 5.3	0.28
PNI under-nutrition present/absent (%)	14	15	0.66
GNRI score	111.1 ± 10.4	111.7 ± 10.8	0.44
GNRI under-nutrition present/absent (%)	9	10	0.54
Creatinine/cystatin C ratio (number)	9.59; (8.15 ÷ 11.48)	10.69; (9.13 ÷ 12.59)	<0.001
GLIM under-nutrition present/absent	39	32	0.05
**Laboratory tests**	Means ± standard deviation/medians with 25 and 75 percentiles or per cent where appropriate		
Hemoglobin (mmol/L)	8.7 ± 1.1	8.7 ± 1.0	0.58
NTproBNP (pg/mL)	1662; (925 ÷ 3846)	1363; (620 ÷ 2981)	0.04
Creatinine (µmol/L)	1.0; (0.8 ÷ 1.3)	0.97; (0.8 ÷ 1.2)	0.11
eGFR_MDRD_ (mL/min × 1.73 m^2^)	83; (62 ÷ 106)	86; (68 ÷ 103)	0.86
Sodium (mmol/L)	136; (134 ÷ 138)	136; (134 ÷ 138)	0.02
hCRP (mg/dL)	2.8; (1.3 ÷ 6.5)	2.7; (1.2 ÷ 6.8)	0.52
**Comorbidities**			
Hypertension (%)	58	53	0.25
Diabetes mellitus type 2 (%)	30	29	0.86
Hypercholesterolemia (%)	62	60	0.57
Hypertriglicerydemia (%)	44	42	0.72
History of smoking (%)	73	74	0.65
**Therapy**			
ACEI/ARB (% treated)	92	95	0.29
ACEI/ARB (% of recommended dose)	50 (20 ÷ 100)	50 (25 ÷ 100)	0.81
BB (% treated)	98	97	0.51
BB (% target of recommended dose)	50 (33 ÷ 66)	50 (33 ÷ 66)	0.45
MRA (% treated)	94	95	0.36
MRA (% of recommended dose)	50 (50 ÷ 100)	50 (50 ÷ 50)	0.13
Loop diuretics (% treated)	91	90	0.59
Loop diuretics (mg of furosemide eq.)	80 (40 ÷ 120)	80 (40 ÷120)	0.11
Mortality at 1 year (%)	15	10	0.06

Legend (Table 2): ICM—ischemic aetiology, NYHA—New York Heart Association, MVO_2_—maximal oxygen consumption during treadmill exercise limited by symptoms of breathlessness, LVEF—left ventricle ejection fraction, PreHF BMI—BMI before HF onset, ASMI—Appendicular skeletal muscle index, CONUT—Controlling Nutritional Status, PNI—Prognostic Nutritional Index, GNRI—Geriatric Nutritional Risk Index, GLIM—Global Leadership Initiative on Malnutrition, NTproBNP—N-terminal propeptide of Brain-type natriuretic peptide, HsCRP—high sensitivity C-reactive protein, ACEI/ARB-—Angiotensin converting enzyme inhibitor or Angiotensin receptor blocker, BB—Beta blocker, and MRA—Mineralocorticoid receptor antagonist.

**Table 3:** Analysis of determinants of low SUCR (N = 721).

Feature	All N = 721		
	Groups of SUCR Defined Based on ROC Analysis		
	SUCR < 0.628 g/L (1 Year Mortality Group)		
	Univariate	Multivariable	
		Model 1	Model 2
	Odds Ratio ± 95% Confidence Interval, *p*-Value		
Male versus female	1.54; (1.06–2.33), *p* = 0.02		
NYHA class (per 1 class increase)	1.46; (1.20–1.77), *p* = 0.0001		
Systolic BP (per 5 mmHg increase)	0.94; (0.89–0.98), *p* = 0.008		
Index BMI (per 1 kg/m^2^ increase)	1.06; (1.3–1.1), *p* = 0.0006		
Weight loss from preHF BMI until index BMI (per 5% increase)	1.13; (1.06–1.20), *p* = 0.0002		
Catabolic component of weight trajectory (per 5% increase)	1.19; (1.10–1.29), *p* < 0.0001		1.43; (1.04–1.97), *p* = 0.03
Catabolic/anabolic balance (per 5% more anabolic)	0.89; (0.83–0.95), *p* = 0.0003		
Fat mass (per 1 kg/m^2^ increase)	1.06; (0.99–1.12); *p* = 0.068		
Fat-free mass (per 1 kg/m^2^ increase)	0.94; (0.89–1.00), *p* = 0.057		
GNRI score (per 5 points increase)	0.95; (0.88–1.01), *p* = 0.120	0.83; (0.72–0.97), *p* = 0.01	0.83; (0.72–0.77), *p* = 0.02
Creatinine/cystatin ratio (per 1 decrease)	1.16; (1.09–1.23), *p* < 0.0001	1.19; (1.10–1.29), *p* < 0.0001	1.18; (1.09–1.28), *p* < 0.0001
GLIM under-nutrition present versus absent	1.41; (1.03–1.93), *p* = 0.030		
NTproBNP (per 1000 pg/mL increase)	1.07; (1.02–1.13), *p* = 0.060		
Sodium (per 5 mmol/L increase)	0.69; (0.57–0.83), *p* < 0.0001		
MRA (% of recommended dose)	1.03; (1.01–1.05), *p* = 0.02		
Loop diuretics (mg of furosemide eq.)	1.11; (1.03–1.19), *p* = 0.004		

Legend (Table 3): Model 1—variables included in the multivariable model: gender (categorical), NYHA class (continues), systolic blood pressure, Index BMI, fat mass, GNRI score (continues), GLIM (categorical), NTproBNP, creatinine/cystatin C ratio, sodium, diabetes (categorical), % recommended MRA, and loop diuretics. Model 2—variables included in the multivariable model: gender (categorical), NYHA class (continues), systolic blood pressure, Index BMI, catabolic component of weight trajectory, % weight loss, catabolic/anabolic balance, fat mass, fat-free mass, GNRI score (continuous), NTproBNP, creatinine/cystatin C ratio, sodium, diabetes (categorical), % recommended MRA, and loop diuretics.
